# Erratum: Oh, E., et al. Cryopreserved Human Natural Killer Cells Exhibit Potent Antitumor Efficacy against Orthotopic Pancreatic Cancer through Efficient Tumor-Homing and Cytolytic Ability (Running Title: Cryopreserved NK Cells Exhibit Antitumor Effect). *Cancers* 2019, *11*, 966

**DOI:** 10.3390/cancers12113255

**Published:** 2020-11-04

**Authors:** Eonju Oh, Bokyung Min, Yan Lin, ChunYing Lian, JinWoo Hong, Gyeong-min Park, Bitna Yang, Sung Yoo Cho, Yu Kyeong Hwang, Chae-Ok Yun

**Affiliations:** 1 Department of Bioengineering, College of Engineering, Hanyang University, 222 Wangsimni-ro, Seongdong-gu, Seoul 04763, Korea; djswn1111@hanyang.ac.kr (E.O.); liyan81@naver.com (Y.L.); liancy100788@daum.net (C.L.); jhong803@gmail.com (J.H.); 2 GeneMedicine Co., Ltd., Seoul 04763, Korea; 3 GC LabCell 107, Ihyeon-ro 30beon-gil, Giheung-gu, Yongin-si, Gyeonggi-do 16924, Korea; bkmin@greencross.com (B.M.); gyeongmin@greencross.com (G.-m.P.); yangbn@greencross.com (B.Y.); chosy@greencross.com (S.Y.C.); 4 Institute of Nano Science and Technology (INST), Hanyang University, 222 Wangsimni-ro, Seongdong-gu, Seoul 04763, Korea

There is an error in the title of the paper [1]. The authors thus wish to make the following correction to this paper [1]:

Change the title from “Cryopreserved Human Natural Killer Cells Exhibit Potent Antitumor Efficacy against Orthotopic Pancreatic Cancer through Efficient Tumor-Homing and Cytolytic Ability (Running Title: Cryopreserved NK Cells Exhibit Antitumor Effect)” to “Cryopreserved Human Natural Killer Cells Exhibit Potent Antitumor Efficacy against Orthotopic Pancreatic Cancer through Efficient Tumor-Homing and Cytolytic Ability”. 

We apologize for this error and state that the scientific conclusions are unaffected. The original article has been updated. 
